# A Simple, Label-Free, and High-Throughput Method to Evaluate the Epigallocatechin-3-Gallate Impact in Plasma Molecular Profile

**DOI:** 10.3390/ht9020009

**Published:** 2020-04-09

**Authors:** Rúben Araújo, Luís Ramalhete, Helder Da Paz, Edna Ribeiro, Cecília R.C. Calado

**Affiliations:** 1 ISEL—Instituto Superior de Engenharia de Lisboa, Instituto Politécnico de Lisboa, 1959-007 Lisboa, Portugal; rubenalexandredinisaraujo@gmail.com (R.A.); luis.m.ramalhete@gmail.com (L.R.); dapazhelder.hdp@gmail.com (H.D.P.); 2 CSTL-T- Centro de Sangue e da Transplantação- Instituto Português do Sangue e Transplantação, IP, 1769-001 Lisboa, Portugal; 3 H&TRC- Health & Technology Research Center, Escola Superior de Tecnologia da Saúde, Instituto Politécnico de Lisboa (ESTeSL), 1990-096 Lisboa, Portugal; edna.ribeiro@estesl.ipl.pt; 4 CIMOSM, Centro de Investigação em Modelação e Optimização de Sistemas Multifuncionais, ISEL, 1959-007 Lisboa, Portugal

**Keywords:** high-throughput, FTIR spectroscopy, EGCG, plasma

## Abstract

Epigallocatechin-3-gallate (EGCG), the major catechin present in green tea, presents diverse appealing biological activities, such as antioxidative, anti-inflammatory, antimicrobial, and antiviral activities, among others. The present work evaluated the impact in the molecular profile of human plasma from daily consumption of 225 mg of EGCG for 90 days. Plasma from peripheral blood was collected from 30 healthy human volunteers and analyzed by high-throughput Fourier transform infrared spectroscopy. To capture the biochemical information while minimizing the interference of physical phenomena, several combinations of spectra pre-processing methods were evaluated by principal component analysis. The pre-processing method that led to the best class separation, that is, between the plasma spectral data collected at the beginning and after the 90 days, was a combination of atmospheric correction with a second derivative spectra. A hierarchical cluster analysis of second derivative spectra also highlighted the fact that plasma acquired before EGCG consumption presented a distinct molecular profile after the 90 days of EGCG consumption. It was also possible by partial least squares regression discriminant analysis to correctly predict all unlabeled plasma samples (not used for model construction) at both timeframes. We observed that the similarity in composition among the plasma samples was higher in samples collected after EGCG consumption when compared with the samples taken prior to EGCG consumption. Diverse negative peaks of the normalized second derivative spectra, associated with lipid and protein regions, were significantly affected (*p* < 0.001) by EGCG consumption, according to the impact of EGCG consumption on the patients’ blood, low density and high density lipoproteins ratio. In conclusion, a single bolus dose of 225 mg of EGCG, ingested throughout a period of 90 days, drastically affected plasma molecular composition in all participants, which raises awareness regarding prolonged human exposure to EGCG. Because the analysis was conducted in a high-throughput, label-free, and economic analysis, it could be applied to high-dimension molecular epidemiological studies to further promote the understanding of the effect of bio-compound consumption mode and frequency.

## 1. Introduction

Tea, derived from the leaves of *Camellia sinensis*, is the second most consumed beverage in the world, losing only to water, with a growth rate of 4% per year, and with predicted sales values of around 7.9 billion US dollars [1]. All tea comes from the leaves of *Camellia sinensis*, but depending on the oxidation and fermentation techniques, four major types of tea (white, green, Oolong, and black) are known. Black tea accounts for 80% of production, whereas green tea makes up for almost the majority of the remaining 20% [2]. Green tea is produced from the unfermented leaves and reportedly contains the highest concentration of antioxidants [3]. Due to green tea’s perceived economic value and health benefits, its production increased 5.4% over the last decade, with respect to an increase of only 3% for black tea. World tea production levels has a predicted yearly increase up to 2027, with 2.2% for black tea and 7.5% for green tea [2]. 

A wide range of health benefits has been associated with the regular consumption of green tea, including anticancer, antidiabetic, antiobesity, anti-inflammatory, and antimicrobial effects, and even effects against some cardiovascular diseases [4]. These bioactivities have been attributed mostly to catechins, the main polyphenol present in tea, making up 32%–40% of the composition of green tea and 10%–12% of black tea per solids [5]. The most abundant catechin in tea, representing between 50% and 80% of these compounds, is epigallocatechin-3-gallate (EGCG) [5]. The widely renowned biological actions of catechins have been associated with their antioxidative properties [6,7].

A typical 240 mL green teacup, prepared from a 2.4g leaf, has an estimated 142 mg of EGCG per cup. Due to its popular health benefits, green tea extracts and even EGCG are commonly used as food supplements, acting as an integral ingredient on many cultures and traditional dishes. The wider use of EGCG as a food supplement has led to its extensive study, and some studies have alerted to its possible toxicological effects on the liver [8]. However, the European Food Safety Authority (EFSA) pointed out that, in general, the consumption of green tea infusions or other ready-to-drink green tea beverages are generally safe, as long as their quantity is kept in check [9]. Concerning the widespread use of supplements, a general warning against doses of EGCG higher than 800 mg/day, which could lead to liver damage, has been issued. In the review conducted by Hu et al. [8], it was indicated that a safe consumption of EGCG obtained from tea beverages was 704 mg dry weight per day and for solid ingestions (e.g., as supplements) was 338 mg dry weight per day. There are diverse studies pointing out the effect of EGCG on in vitro cell cultures or in vivo based on animal models, as reviewed, for example, in Khan and Mukhtar [5]. Human studies focus, on the majority, on epidemiological evaluations, and few are based on clinical data from conventional blood analysis, as conducted over cancer development and progression [10], metabolic syndrome [11], and cardiovascular diseases [12,13]. Human studies using catechins as a supplement observed a decrease of plasma Low-Density Lipoproteins (LDL) in comparison with controls, and in smokers a reduction of benzo[a]pyrene adducts [14,15,16].

The present work aimed to evaluate the impact of EGCG consumption on the plasma molecular profile as obtained from high-throughput Fourier transform infrared (FTIR) spectroscopy, a very sensitive and specific technique. Examples of the technique’s high sensitivity can be observed in applications to discriminate similar cells as bacteria serogroups from *Salmonella* [17], stem cell differentiation [18], cancer diagnosis and cancer grading [19,20], and to conduct diagnosis and prognosis of a diverse range of other diseases and the monitoring of treatments efficiency [21,22,23]. The high versatility of this technique also enables its application to a high diversity of biological samples, from tissue-based biopsies to body fluids such as urine, saliva, and tears [23,24,25]. 

FTIR spectroscopy on the mid-infrared region of the spectra (between 4000 and 400 cm^−1^) covers molecular fundamental vibrations of most common biomolecules. There are three main spectral regions that mainly reflect bond stretching and bond deformations: between 3600 and 2000 cm^−1^, mostly due to vibrations between X–H (where X can be C, O, or N), as present in amide A (≈3200 cm^−1^) of proteins, CH_3_ (≈2955 and ≈2870 cm^−1^) and CH_2_ (≈2918 and ≈2850 cm^−1^) groups from lipids; 1800–1500 cm^−1^, mostly due to double bonds as C=O, C=C, and C=N, as present in amide I (≈1650 cm^−1^) and amide II (≈1550 cm^−1^) of proteins, C=O as from phospholipids esters (≈1740 cm^−1^); and 1500–400 cm^−1^, known as the fingerprint region, due to overlapped vibrations from diverse molecules [26,27]. 

This vibrational spectroscopic technique is simple to apply, with a sample requiring for most cases a simple pre-processing step such as dehydration. It is economic, as no expensive reagents are needed and it boast a rapid workflow, as one spectra is usually acquired in 1 min [26,27,28]. Furthermore, the technique also presents diverse modes of detection, from transmission, transflectation, and attenuated total reflection. In the present work, it was selected as a detection mode on the basis of transmission analysis conducted in a high-throughput mode, by using microplates with 96 micro-wells [26,27,28,29].

A diverse range of authors have conducted a uni-variate analysis of the spectrum, focusing their analysis on defined peaks, ratios of peaks, or its derivatives, in order to define a specific biological phenotype or even a metabolic interpretation [27,28,29]. However, for a more efficient extraction of features and information in complex spectra, as it is the case for plasma spectra, multivariate data analysis is usually the tool of choice. Indeed, due to the complexity of biological samples such as plasma, different molecules share common bonds, and consequently share the same spectral regions, making it difficult to attribute to a single biomolecule. For example, the bands of ≈1084 and ≈1245 cm^−1^ are usually attributed to symmetric and antisymmetric vibrations of phosphate bonds, respectively, which are present in diverse molecules such as DNA, RNA, phospholipids, and phosphorylated proteins [28,30]. Principal component analysis (PCA) is the most frequently used qualitative multivariate data analysis technique, as it allows for dimension reduction while enabling a search for data patterns, whereas other methods such as hierarchical clustering analysis (HCA) and supervised discriminant analysis (DA) [31,32], allow for the quantitative analysis and the development of prediction models, respectively. 

In our previous and preliminary work, we observed that the plasma FTIR spectra, obtained from 30 human participants, presented a distinct profile after 90 days of EGCG consumption [33]. We observed, via PCA of plasmas’ spectra, a very good separation of the data clusters on the score plots between plasma samples before and after the 90 days of EGCG consumption. On the basis of the PCA loadings, it was possible to note spectral regions associated to the effect of EGCG on the plasma molecular profile, and with it identify ratios of spectral peaks that were statistically different, between spectra acquired before and at the end of 90 days of EGCG consumption. The present study complements this work by evaluating other spectra pre-processing techniques and quantitative unsupervised and supervised multivariate data analysis such as by HCA and DA. We intended to promote a new methodology, based on high-throughput FTIR spectroscopy associated with univariate and multi-variate data analysis, applicable in higher dimension studies, and consequently enabling a better and more comprehensive knowledge of the effect of biocompounds on the human physiological state. 

## 2. Materials and Methods

### 2.1. Biological Assay

The study included 30 healthy individuals (10 males and 20 females) between 18 and 45 years of age. Volunteers provided a signed written informed consent before enrolment in the study, with all the inherent ethical principles properly safeguarded.

Peripheral blood was collected with anticoagulant ethylenediaminetetraacetic acid (EDTA) according to standard blood collection procedures, before (T0) and after 90 days (T90) daily consumption of a green tea capsule with 225mg EGCG. Plasma was obtained by centrifuging fresh blood sample at 3500 rpm for 10 min (Mikro with 1195/L rotor, Hettich, Tuttlingen, Germany).

### 2.2. FTIR Spectra Acquisition

In general, triplicates of 25 μL of plasma, diluted at 1/10 in water, from each volunteer at T0 and T90 were transferred to a 96-well Si (Bruker, Germany) plate and then dehydrated for about 2.5 h in a desiccator under vacuum (Vacuubrand, ME 2, Wertheim, Germany). Due to limitations of sample quantity, the spectra of the sample T90 of the first volunteer was conducted only in duplicate. 

Spectral data were collected using a FTIR spectrometer (Vertex 70, Bruker, Germany) equipped with a microplate reader extension for high-throughput screening in infrared spectroscopy, a module accessory designated as HTS-XT (Bruker, Ettlingen, Germany). Each spectrum represented 64 coadded scans, with a 2cm^−1^ resolution, and were collected in transmission mode between 400 and 4000 cm^−1^. The first well of the 96-well plate did not contain a sample, and the corresponding spectra were acquired and used as background, according to the HTS-XT manufacturer. 

### 2.3. Spectra Pre-Processing and Processing

Spectra were pre-processed by atmospheric correction, baseline correction, unit vector normalization, standard normal variate (SNV), multiplicate scatter correction (MSC), and its extended version (EMSC). First and second derivative spectra were computed from raw spectra pre-processed only with atmospheric correction, using a Savitzky–Golay filter, and a second order polynomial over a 15-point window. Atmospheric and baseline corrections were conducted with OPUS software, version 6.5 (Bruker, Ettlingen, Germany), and all remaining pre-processing work, PCA, HCA (based on Spearman’s rank correction and hierarchical average-linkage), and partial least squares regression associated with discriminant analysis (PLS-DA) were conducted with The Unscrambler X, version 10.5 (CAMO software AS, Oslo, Norway). The statistical analysis by Student’s *t*-test was conducted on the basis of peak height of the second derivative spectra with vector unit normalization, between 400 and 1800 and 2700 and 4000 cm^−1^. The Student’s *t*-test was based on a paired test with two tails, and was performed on Microsoft Excel.

## 3. Results and Discussion

**Figure 1:**
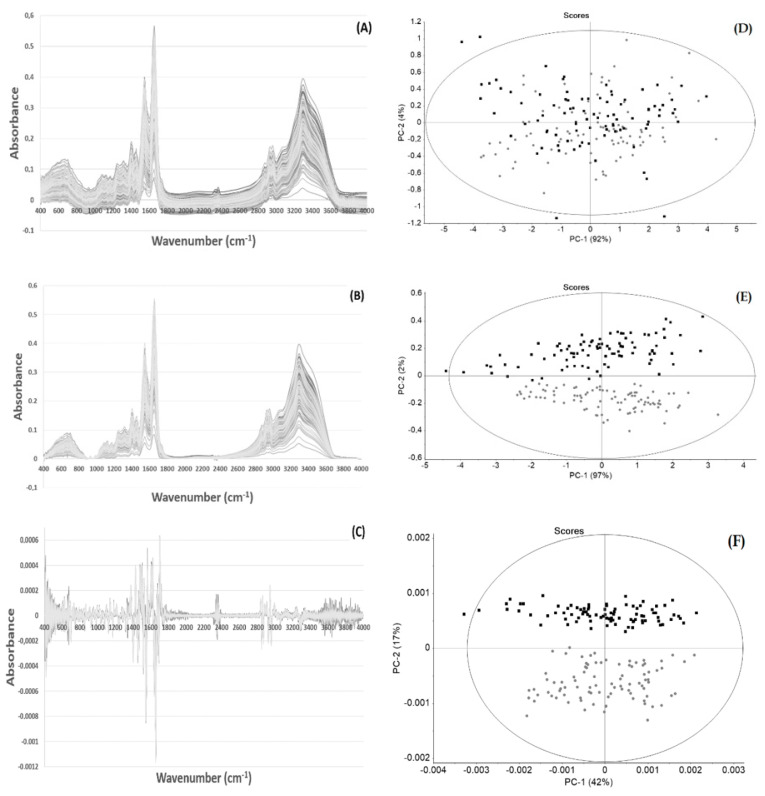
Triplicates of plasma spectra obtained before (T0, circle, grey) and after 90 days of epigallocatechin-3-gallate (EGCG) consumption (T90, square-shaped, black), with diverse pre-processing techniques (**A**,**B**,**C**) and the corresponding principal component analysis (PCA) score-plots (**D**,**E**,**F**), respectively. Spectra with no pre-processing (**A**,**D**), atmospheric and baseline correction (**B**,**E**), and with atmospheric correction and second derivative (**C**,**F**). PCA presents the Hotelling’s ellipse at 1% significance.

Previous human clinical trials established that 400 to 800 mg of EGCG is considered a safe dose for human consumption [34]. This dose resulted in peak serum concentrations that ranged from 100 to 400 ng/mL [35]. In a previous work of the current research team, it was observed that an intake of 225 mg of EGCG/day for 90 days affected the following, as shown from blood analysis: erythrocytes, hemoglobin, hematocrit, mean cell volume, reticulocyte hemoglobin content, fetal hemoglobin level, and the ratio between LDL and High-Density Lipoproteins (HDL) [33]. In that work, it was also observed that EGCG consumption affected the plasma FTIR spectra, because samples obtained before EGCG consumption grouped together and apart from samples taken after 90 days of EGCG consumption on a PCA score-plot. In the present work, and in order to quantify the impact of EGCG consumption on the plasma molecular profile, an HCA and a prediction model based on PLS-DA were developed. Accordingly, it the effect of several spectra pre-processing methods was evaluated in order to minimize the effect of physical distortions (as due to light scattering), while enhancing the sample chemical characteristics inherent to each spectrum. The effect of spectra pre-processing was evaluated on PCA score plot, as it facilitates data visualization for a better insight into the identification of major trends. Diverse combinations of the following spectra pre-processing methods were evaluated: atmospheric and baseline correction; normalization; SNV, MSC, and EMSC; and first and second derivatives. Among them, the pre-processing methods that resulted in better data separation between samples taken before and after EGCG consumption on the PCA sore plot are represented in Figure 1. All included atmospheric correction that minimized the spectra interference due to atmospheric water and carbon dioxide. The use of atmospheric correction, followed by second derivative, led to the best sample separation (Figure 1), by which PC1 and PC2 included 59% of data variance. In the following work, this was the pre-processing method used. In all pre-processing methods, only one sample was found to be outside of the Hotelling’s ellipse at 1%. This replica of triplicate analysis was then considered an outlier of the analysis process because the remaining two replicas were inside the Hotelling’s ellipse. 

**Figure 2:**
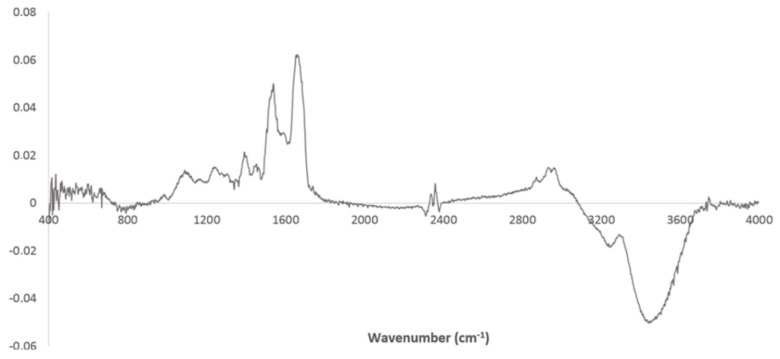
Loadings of PC2 of PCA conducted with atmospheric and baseline correction.

PC2 loadings of PCA based on spectra pre-processed by atmospheric and baseline correction (Figure 2) highlight spectral regions that contributed to sample separation between T0 and T90. Therefore, the spectral regions of plasma most affected by EGCG consumption were 400–600, 1000–1800, and 2800–3700 cm^−1^, pointing out relevant regions including amide A, B, I, and II from proteins (at 3300, 1656, and 1548 cm^−1^); CH_3_ and CH_2_ from lipids (e.g., between 2800–3000 cm^−1^); phosphate groups from lipids and proteins (e.g., 1287 and 1082 cm^−1^); and regions in the fingerprint. This points to a high impact of EGCG consumption on plasma whole molecular composition, and consequently on the general cellular metabolism. These observations were in accordance with the effect of EGCG consumption on the blood clinical analysis, as pointed out above, and on cell metabolism as on glycolysis, pentose phosphate pathway, serine biosynthesis, mitochondria energetic metabolism and lipid metabolism, and lipid peroxidation [36,37,38,39]. The high impact of the region between 3200 and 4000 cm^−1^ includes the amide A amide from proteins, according to Liu et al. [40], who included this region in PLS regression models to predict (LDL from serum spectra. 

**Figure 3:**
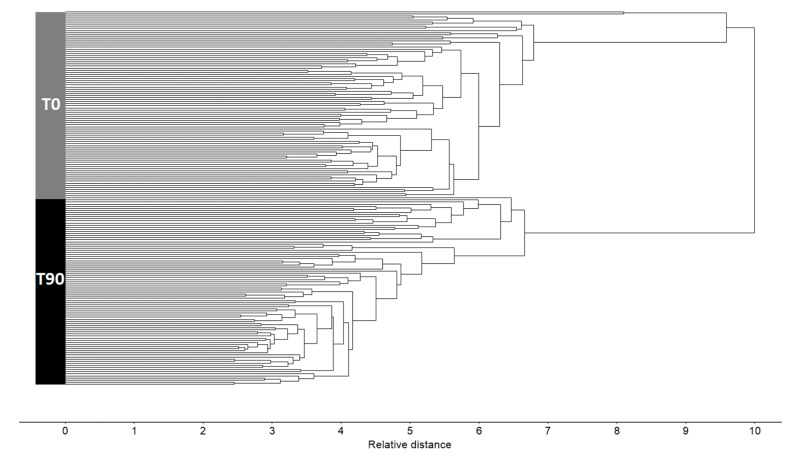
Hierarchical clustering analysis (HCA) of triplicates of plasma spectra obtained before (T0, grey) and after 90 days of EGCG consumption (T90, black), pre-processed with atmospheric correction and second derivative.

HCA was used to evaluate the intrinsic data patterns in plasma spectra and to determine pairwise distances that were visualized in hierarchical trees (i.e., dendrograms), which served to evaluate the variability between different classes (T0 and T90). We observed a complete separation between the two classes of plasma spectra (Figure 3). Interestingly, the HCA dendrogram pointed out the fact that samples taken before EGCG consumption apparently presented a higher variability between them than samples taken after EGCG consumption. 

**Figure 4:**
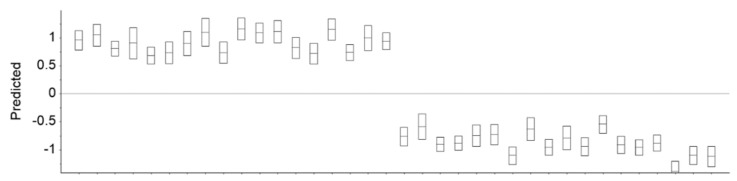
Prediction and respective deviation if a sample was taken before or after EGCG consumption in relation to real measurements taken before (-1) or after 90 days (+1) of EGCG consumption. Triplicate samples from six patients taken at two time frames were considered.

PLS-DA was applied as a supervised classification method in order to evaluate whether differences between plasma spectra recorded before and after EGCG consumption were high enough to enable to predict from a spectrum when the sample was collected (i.e., before or after EGCG consumption). The prediction model was built on the basis of 80% of samples classified as belonging to T0 or T90 (i.e., triplicate data from 24 patients). The model, built on three factors, was then used to evaluate the classification of the remaining samples (that were not used in model building). In general, all the validation samples were correctly classified, presenting prediction values near −1 and +1 on the basis of whether they were taken before or after EGCG consumption, respectively (Figure 4). Interestingly, all samples taken after EGCG consumption presented a prediction value with its deviation higher than 0.5, and consequently were predicted with confidence. Of the validation samples taken before EGCG consumption, 3 out of 18 showed prediction values with deviation lower than −0.5, leading to lower prediction confidence. These three samples were each from the triplicate analyses from patients 25, 27, and 28. The other two duplicates of each patient presented values (considering the deviation) lower than −0.5, and consequently were well predicted. This highlights the importance of conducting replicate analysis due to high sensitivity of FTIR spectroscopy. According to above observations, the root square mean error of prediction for samples taken before and after EGCG consumption were 23% and 18%, respectively. This corroborates the apparent higher variability among samples taken before EGCG consumption in relation to samples taken after EGCG consumption, as previous observed by HCA. 

**Figure 5:**
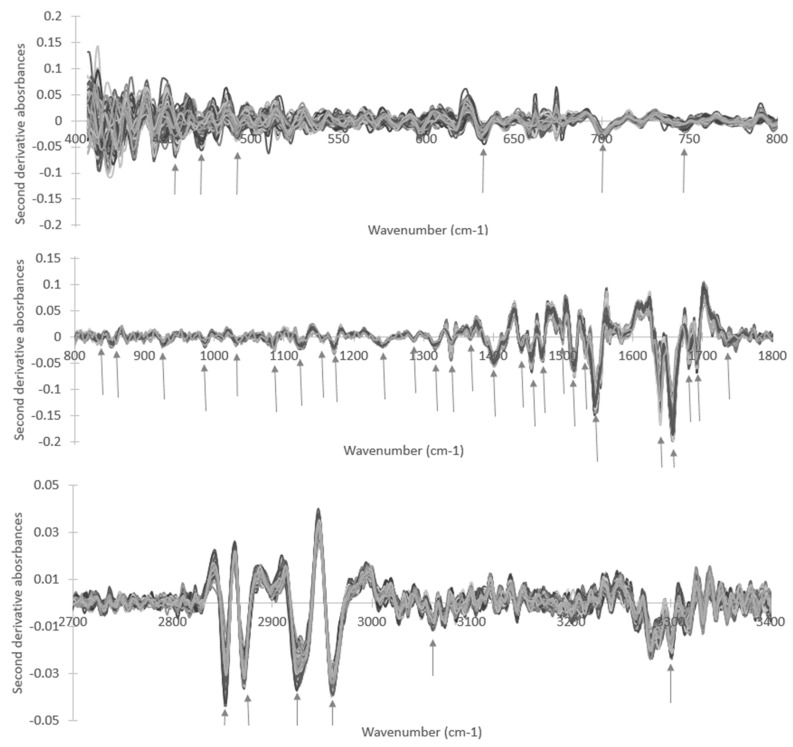
Plasma second derivative spectra with vector unit normalization spectra, highlighting peaks (by arrows) selected for subsequent Student’s *t*-test analysis.

To evaluate the most relevant spectral regions that discriminate plasma samples between T0 and T90, the negative peaks of a set of second derivative spectra were considered, which were obtained from six samples chosen randomly. The second derivative was considered because it resolved superimposed peaks while resulting in sharper peaks, which were easier to identify and consequently to analyze. The negative peaks were considered because they represented positive peaks in a non-derivative spectrum. To confirm that the peaks on the derivative spectra were not due to noise amplification, a set of six non-derivatised spectra randomly chosen were also considered. The negative peak of the second derivative spectra should be present in these six non-derivatized spectra as a peak or a shoulder. On the basis of this, a total of 39 peaks were identified, as represented by arrows in Figure 5. A paired Student’s *t*-test was then applied to compare the peak heights from the second derivative spectra with unit vector normalization between triplicate spectra of all 30 volunteers acquired before and after EGCG consumption, that is, between T0 and T90, respectively. To conduct a paired *t*-test, only duplicate samples of the first volunteer were considered, as only duplicate spectra were acquired for T90 due to a limitation of the sample quantity. One of the triplicate spectra analyses of the third volunteer at T0 and T90 was also discarded, as one spectrum from the triplicates acquired at T90 was identified as an outlier in the PCA represented in Figure 1F.

**Figure 6:**
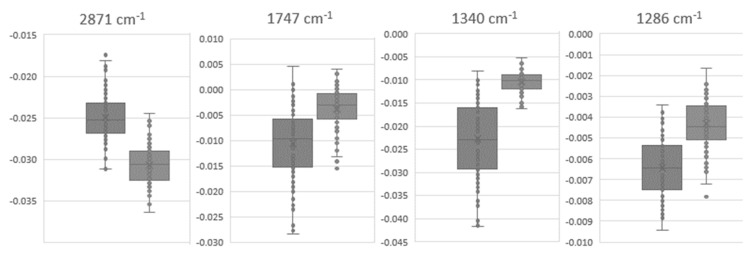
Values of replicate analysis of second derivative absorbances with vector unit normalization at 1747, 1340, 1286, and 933 cm^−1^ of the plasma samples of 30 volunteers at T0 (left panel) and T90 (right panel).

From the 39 peaks evaluated, 28 presented values statistically different (*p* < 0.001) (Table 1), which included bond vibrations in amides A, B, I, and II from proteins at 3300, 3070, 1657, 1639, 1544, and 1515 cm^−1^; diverse bond vibrations from lipids, such as from CH_3_ and CH_2_ at 2871, 2961, 1469, 1440, 1401, 1340, and 1317 cm^−1^ and from C=O at 1691 and 1746 cm^−1^; bond vibrations on phosphate groups present in lipids and phosphoproteins such as at 1286, 1241, and 1083 cm^−1^; C-OH from carbohydrates such as from 1172, 1156, and 1122 cm^−1^ from nucleic acids and at 1032 cm^−1^ from glycogen; and diverse peaks at the fingerprint region (Table 1). Boxplots of some of these peaks that resulted in the lowest *p*-values are represented in Figure 6. The high impact on the lipids and protein bands were on the basis of the effect of EGCG consumption on blood LDH/HDL (*p* < 0.05).

## 4. Conclusions

EGCG consumption led to a statistically significant difference in the plasma whole molecular composition, as acquired by simple, label-free, and high-throughput FTIR spectroscopy. This impact was observed in HCA, where spectra from all plasma samples acquired before and after EGCG consumption were in distinct clusters. The high impact of EGCG consumption on plasma composition enabled the development of prediction models, based on PLS-DA, making it possible to predict from the plasma spectrum whether the sample was from a person that consumed EGCG. The HCA and PLS-DA also pointed out that plasma composition from individuals presented a more similar composition among them after EGCG consumption, probably due to EGCG effect on blood composition, as well as on LDL/HDL. In terms of means of the population, we observed statistically significant (*p* < 0.001) differences at diverse spectral peaks (associated with proteins, lipids, phosphate groups, and carbohydrates) between before and after EGCG consumption. This significant impact of EGCG consumption on the plasma signature raises awareness regarding the potential human health effects from prolonged consumption/exposure to EGCG, reinforcing the need for further studies. The methodology applied enabled, therefore, the ability to quantify the impact of EGCG consumption on plasma whole molecular composition by a simple, economic, label-free, rapid, and high-throughput mode on the basis of a simple drop (i.e., 10 µL) of blood. This methodology can be applied in large scale epidemiological studies, enabling a better understanding of the impact of a bio-compound (such as EGCG) consumption mode, such as quantities, frequencies, and interferences with other food components, as well as its relationship with a persons’ health and lifestyle. 

## Figures and Tables

**Table 1:** Average values and standard deviations of plasma spectra peaks (based on second derivative and unit vector normalization) at T0 and T90, respectively, and *p*-value of Student’s *t*-test regarding the comparison of the T0 population in relation to the T90 population, at *p* < 0.001.

Wavenumber (cm^−1^)	T0		T90		*p*-Value (T0 vs. T90)
	Average	Standard Deviation	Average	Standard Deviation	
3331	−1.30 × 10^−3^	2.03 × 10^−3^	−3.87 × 10^−3^	2.05 × 10^−3^	1.89 × 10^−13^
2961	−3.15 × 10^−2^	2.49 × 10^−3^	−3.26 × 10^−2^	2.55 × 10^−3^	7.37 × 10^−3^
2872	−2.50 × 10^−2^	2.89 × 10^−3^	−3.07 × 10^−2^	2.31 × 10^−3^	6.85 × 10^−23^
1747	−1.06 × 10^−2^	7.29 × 10^−3^	−3.77 × 10^−3^	4.12 × 10^−3^	1.93 × 10^−14^
1691	−3.53 × 10^−2^	8.19 × 10^−3^	−4.44 × 10^−2^	8.63 × 10^−3^	9.60 × 10^−12^
1658	−1.70 × 10^−1^	2.00 × 10^−2^	−1.59 × 10^−1^	1.98 × 10^−2^	2.38 × 10^−4^
1639	−8.81 × 10^−2^	1.99 × 10^−2^	−1.00 × 10^−1^	2.87 × 10^−2^	1.63 × 10^−3^
1545	−1.27 × 10^−1^	1.17 × 10^−2^	−1.17 × 10^−1^	1.32 × 10^−2^	7.05 × 10^−7^
1516	−4.75 × 10^−2^	7.44 × 10^−3^	−5.75 × 10^−2^	8.61 × 10^−3^	6.71 × 10^−13^
1470	−3.55 × 10^−2^	3.77 × 10^−3^	−3.77 × 10^−2^	4.42 × 10^−3^	1.59 × 10^−3^
1441	−1.70 × 10^−2^	4.83 × 10^−3^	−2.19 × 10^−2^	3.32 × 10^−3^	1.97 × 10^−13^
1401	−4.47 × 10^−2^	4.63 × 10^−3^	−4.89 × 10^−2^	2.88 × 10^−3^	2.77 × 10^−11^
1340	−2.28 × 10^−2^	8.56 × 10^−3^	−1.04 × 10^−2^	2.18 × 10^−3^	6.02 × 10^−25^
1317	−1.95 × 10^−2^	2.56 × 10^−3^	−1.45 × 10^−2^	1.48 × 10^−3^	3.61 × 10^−27^
1286	−6.46 × 10^−3^	1.44 × 10^−3^	−4.32 × 10^−3^	1.15 × 10^−3^	1.10 × 10^−15^
1241	−1.53 × 10^−2^	1.47 × 10^−3^	−1.64 × 10^−2^	1.29 × 10^−3^	3.12 × 10^−6^
1173	−2.37 × 10^−2^	2.67 × 10^−3^	−2.14 × 10^−2^	3.79 × 10^−3^	2.38 × 10^−5^
1156	−1.57 × 10^−3^	1.69 × 10^−3^	−3.98 × 10^−3^	2.00 × 10^−3^	3.28 × 10^−13^
1122	−1.35 × 10^−2^	3.52 × 10^−3^	−1.59 × 10^−2^	2.80 × 10^−3^	1.86 × 10^−6^
1084	−1.19 × 10^−2^	2.39 × 10^−3^	−1.35 × 10^−2^	1.79 × 10^−3^	5.28 × 10^−7^
1032	−1.10 × 10^−2^	2.05 × 10^−3^	−9.63 × 10^−3^	2.33 × 10^−3^	2.41 × 10^−4^
987	−9.40 × 10^−3^	2.33 × 10^−3^	−1.19 × 10^−2^	2.76 × 10^−3^	2.46 × 10^−27^
933	−1.00 × 10^−2^	1.74 × 10^−3^	−4.49 × 10^−3^	2.69 × 10^−3^	2.46 × 10^−27^
838	−2.26 × 10^−3^	2.13 × 10^−3^	−5.05 × 10^−3^	2.64 × 10^−3^	2.28 × 10^−13^
746	−1.45 × 10^−2^	2.10 × 10^−3^	−9.17 × 10^−3^	2.90 × 10^−3^	6.37 × 10^−26^
634	−1.61 × 10^−2^	4.79 × 10^−3^	−1.85 × 10^−2^	6.11 × 10^−3^	8.33 × 10^−3^
492	−1.04 × 10^−2^	8.28 × 10^−3^	−1.47 × 10^−2^	8.11 × 10^−3^	6.39 × 10^−4^
473	−2.52 × 10^−2^	1.02 × 10^−2^	−1.11 × 10^−2^	1.01 × 10^−2^	6.47 × 10^−13^
